# 0852. Selective decontamination of the digestive tract modulates the metabolic profile in a ventilator-induced lung injury model

**DOI:** 10.1186/2197-425X-2-S1-P61

**Published:** 2014-09-26

**Authors:** Y Rojas, S Naz, JL Izquierdo, N Nin, A Ferruelo, P García-Hierro, D Molina-Arana, R Herrero, L Martínez-Caro, A García, MA de la Cal, JM Ruiz-Cabello, C Barbas, JA Lorente

**Affiliations:** Centro de Investigación Biomédica en Red de Enfermedades RespiratoriaS (CIBERES), Getafe, Spain; Intensive Care Service and Burn Unit, Hospital Universitario de Getafe, Getafe, Spain; Facultad de Farmacia, Centro de Metabolómica y Bioanálisis (CEMBIO), Universidad CEU San Pablo, Madrid, Spain; Centro de Investigaciónes Cardiovasculares (CNIC), Madrid, Spain; Intensive Care Service, Hospital de Torrejon, Madrid, Spain; Intensive Care Service, Hospital Español, Montevideo, Uruguay; Hospital Universitario de Getafe, Getafe, Spain; Universidad Europea de Madrid, Madrid, Spain

## Introduction

Acute lung injury induced by mechanical ventilation [ventilator-induced lung injury (VILI)]is characterized by a particular metabolic profile in the lung and in the systemic compartment [1]. Also, VILI has been associated with an increase in intestinal permeability [2]. We hypothesized that selective decontamination of the digestive tract (SDD) can modulate the metabolic profile associated with mechanical ventilation.

## Objectives

To determine (1) the metabolic profile associated with VILI to identify potential biomarkers; (2) whether SDD modifies this metabolic profile associated with VILI.

## Methods

Rats were pretreated with antibiotics by oral gavage for SDD (polymyxin E 30 mg/ml, tobramycin 12 mg/ml) or vehicle (water) as control. Twenty four hours later, rats were ventilated for 2.5 h. VILI was induced by using high tidal volume (V_T_= 25 ml/kg) + PEEP = 0 cm H_2_O. As control, rats were ventilated with low V_T_ (9 ml/kg) + PEEP = 5 cm H_2_O. We studied four groups: Low V_T_-SDD, High V_T_-SDD, Low V_T_-vehicle and High V_T_-vehicle (n=20 per group). Lung tissue and serum were analyzed by ^1^H-nuclear magnetic resonance spectroscopy (H-MRS) and high pressure liquid chromatography coupled to quadruple time-of-flight (LC- MS-QTOF), respectively. Principal component (PCA) [unsupervisated] and partial least squares (PLS) [supervised] analyses were performed. Accurate masses of features representing significant differences were searched against the MELTING, KEGG, LIPIDMAPDS and HMDB databases. We followed the Principles of Laboratory Animal Care (2010/63/UE 22-09, RD 53/2013 BOE 1-02, ley 32/2007 BOE 7-11).

## Results

We found different metabolic patterns between rats ventilated with low and high V_T_, and also between ventilated rats with and without SDD. In the lung, the main metabolic pathways affected are involved in energy metabolism (creatine, glucose, lactate, alanine, glutamate), protein synthesis (leucine) and membrane lipids (choline, phosphoethanolamine). In serum, the main affected pathways were related to conjugated bile acids, ceramide, Land´s cycle and carnitine biosynthesis.

## Conclusions

(1) Mechanical ventilation can change the metabolic profile in the lung and in the systemic compartment. (2) SDD can modify this metabolic changes induced by mechanical ventilation. (3) Metabolic studies can be useful to identify biomarkers for the diagnosis of acute lung injury, and to design new therapeutic strategies.
